# The Structures of Heterogeneous Membranes and Their Interactions with an Anticancer Peptide: A Molecular Dynamics Study

**DOI:** 10.3390/life12101473

**Published:** 2022-09-22

**Authors:** Ghulam Abbas, Alfredo E. Cardenas, Ron Elber

**Affiliations:** 1 National Center for Bioinformatics, Quaid-i-Azam University, Islamabad 45320, Pakistan or; 2 Oden Institute for Computational and Engineering Sciences, University of Texas at Austin, Austin, TX 78712, USA; 3 Department of Chemistry, University of Texas at Austin, Austin, TX 78712, USA

**Keywords:** heterogeneous membranes, anticancer peptide, molecular dynamics simulations, cell-penetrating peptide

## Abstract

We conduct molecular dynamics simulations of model heterogeneous membranes and their interactions with a 24-amino acid peptide—NAF-1^44–67^. NAF-1^44–67^ is an anticancer peptide that selectively permeates and kills malignant cells; it does not permeate normal cells. We examine three membranes with different binary mixtures of lipids, DOPC–DOPA, DOPC–DOPS, and DOPC–DOPE, with a single peptide embedded in each as models for the diversity of biological membranes. We illustrate that the peptide organization in the membrane depends on the types of nearby phospholipids and is influenced by the charge and size of the head groups. The present study sheds light on early events of permeation and the mechanisms by which an amphiphilic peptide crosses from an aqueous solution to a hydrophobic membrane. Understanding the translocation mechanism is likely to help the design of new permeants.

## 1. Introduction

Biological membranes are thin bilayers with a width of ~40 Å that separate external solutions from the interior of cells. Further, they partition the cellular medium into compartments. They are highly heterogeneous and consist of many types of phospholipids, numerous protein components, and other embedded molecules, such as cholesterols. This heterogeneity is necessary for their function, and it extends beyond the molecular scale. The membrane adapts mesoscopic curvatures and shapes and responds to environmental changes. The rich behavior of these multiscale bilayers makes them fascinating systems to study experimentally and theoretically. Computational studies at multiple scales use atomistic and coarse-grained models [1,2,3,4,5] and continuum theory [6,7,8,9].

The spatial and temporal complexity of bio-membranes is a significant computational challenge. Not only are these systems large and include, in typical simulations, tens to hundreds of thousands of particles, but the equilibration times of their diverse components can be exceptionally long. Experimentally, estimates of time scales for forming heterogeneous microdomains in membranes (so-called rafts [10,11]) vary from microseconds to seconds. Because of their complexity, atomically detailed simulations, which provide comprehensive information on solvent, solutes, and their interactions with membranes, are hard to converge. Algorithms that mix Monte Carlo (MC) moves with straightforward molecular dynamics (MD) simulations [12] improve the convergence considerably (e.g., Molecular Dynamics with Alchemical Steps (MDAS) [13]), but they are limited to membranes with similar components and small modifications of the hydrocarbon chains. MC moves are less successful when the phospholipid head groups are modified. The algorithms that mix MD and MC steps are more general for coarse-grained models in which the differences between the phospholipids are reduced [14]. However, the computational gain of the coarse-grained simulations compared to atomically detailed calculations with MDAS is moderate and about a factor of three.

**Figure 1:**
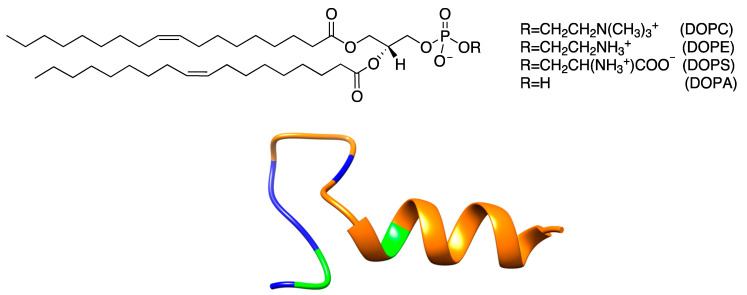
Top: The phospholipids used in the simulations. DOPC—1,2-Dioleoyl-sn-glycero-3-phosphocholine, DOPE—1,2-Dioleoyl-sn-glycero-3-phosphoethanolamine, DOPA—1,2-Dioleoyl-sn-glycero-3-phosphate, DOPS—1,2-Dioleoyl-sn-glycero-3-phosphoserine. Bottom: Ribbon presentation of the backbone of the NAF-1^44–67^ peptide. The N terminus is on the right. The positively charged residues (lysine and arginine) are shown in blue, non-polar residues in orange, and neutral polar residues (tyrosine and glutamine) in green. The N-terminus side of the peptide tends to be helical when embedded in the membrane.

In the present manuscript, we study in atomistic details, using MD simulations, three heterogeneous membranes. Each membrane has two types of phospholipids and a peptide embedded in it (Figure 1). The membranes are mixtures of (1) DOPC and DOPS, (2) DOPC and DOPA, and (3) DOPC and DOPE. The full names of the phospholipids are provided in Figure 1. The selection of the phospholipids leads to membrane heterogeneity that is strictly in the head groups, while the hydrocarbon chains are the same. Applying the combined MD and MC approach, which is most effective for rapidly mixing hydrocarbon chains, is therefore difficult. As a result, we conduct only straightforward MD simulations, and our sampling is limited. Nevertheless, we can use the MD calculations to learn about the differences between the membranes and their interactions with a permeating peptide, at least on the microsecond time scale.

Membranes of a single or a few phospholipid components have been investigated extensively using straightforward MD [15,16,17,18,19]. However, exploring alternative membrane compositions and their interaction with anticancer peptides is new. We focus on the interaction of the peptide NAF-1^44–67^ [20,21,22,23] with different binary mixtures of the above phospholipids. NAF-1^44–67^ is a peptide of 24 amino acids in length (FLGVLALLGYLAVRPFLPKKKQQK). It is positively charged (+5), and its N terminal segment is hydrophobic. It permeates and kills cancer cells; in contrast, it does not permeate or affect normal cells [22]. Therefore, NAF-1^44–67^ is an anticancer agent of considerable biomedical interest. It belongs to a large class of molecules called cell-penetrating peptides (CPP). However, as discussed below, it is unique in its origin and physical interactions with the membrane.

Hundreds of CPPs have been reported with varying properties and significance [24]. Some peptides are highly charged, such as the TAT peptide (+8), and are likely to be unstructured [25]. However, other peptides can have a partial secondary structure, frequently a helix. The helix in this class is amphiphilic, supporting the permeation into biological membranes. The insertion exposes the hydrophilic residues to the aqueous solution and the hydrophobic residues to the lipid environment of the membrane interior [26]. A permeation mechanism in which the helix orients itself parallel to the membrane plane is typical in antibacterial peptides [26].

NAF-1^44–67^ is different from these two common classes. It is a fragment of a stable transmembrane protein that resides in mitochondria [22]. The first eleven residues form an entirely hydrophobic helix. This observation is not surprising for a peptide derived from a transmembrane protein but, to our knowledge, is rare in a CPP. Several CPPs are known to have a leading hydrophobic sequence followed by a charged C terminal segment. These peptides are obtained by covalently attaching hydrophobic residues to a charged peptide to make it soluble in membranes and aqueous solutions.

An example of an amphiphilic CPP is MPG (GLAFLGAAGSTMGAWSQPKKKRKV) [27]. A secondary structure predictor (PROTEUS [28])) suggests that the entire MPG sequence is unstructured. In contrast, the same server predicts the N terminus segment of NAF-1^44–67^ to be helical. The combined distinctive origin, sequence, and secondary structure make NAF-1^44–67^ a unique CPP.

In the full NAF-1 protein, the hydrophobic helix is found inside the membrane with negligible exposure to solvent. The remainder of the peptide is soluble and highly charged (+5). The unexpected observation about NAF-1^44–67^ is not that it permeates membranes, given its origin. Instead, it is that it selectively permeates into cancer cells but does not permeate to the plasma membrane of normal cells. We believe that since it is a fragment of a transmembrane protein, understanding the permeation mechanism of the peptide may shed light on another and not less complex question of the insertion of transmembrane proteins.

Given the high complexity of membranes of living cells, the study of simplified model systems that capture essential differences between the membranes is warranted. The outer layers of plasma membranes of cancer cells are enriched with negatively charged phospholipids compared to the plasma membranes of normal cells [29,30,31]. It is therefore interesting to examine simplified heterogeneous membranes that differ in their charges. The types of phospholipids that we examined include neutral molecules (DOPC and DOPE) and negatively charged head groups (DOPS and DOPA). The comparisons of peptide interactions with different phospholipid mixtures elucidate the molecular mechanism of permeation selectivity. In the process, we also examine the organization, stability, and fluidity of each of the membranes.

The permeation of the anticancer peptide NAF-1^44–67^ into malignant cells but not to normal cells [22] raises the question of membrane selectivity. What is the phospholipid composition that supports translocation across cancer but not normal membranes? In reference [22], we argued that the positively charged NAF-1^44–67^ peptide is attracted to the more negative cancer membrane. However, biological membranes are complex and include many components. It can be challenging to pinpoint a single factor of selectivity. It is therefore of interest to examine simpler compositions and check if, for example, the membrane’s charge has a dominant effect on binding and permeation. Another suggestive factor to examine is the size of the phospholipid heads, which may also affect permeability. We probe simple binary mixtures of charged and neutral phospholipids (e.g., DOPS and DOPC) to mimic the plasma membranes of malignant cells. We also simulate mixtures of neutral phospholipids (e.g., DOPC and DOPE) to model membranes of normal cells. Finally, we looked at charged phospholipids with a small head group (DOPA). We expect that the results of the current simulations will shed light on the operation of more complex biological membranes.

## 2. Methods

The three membrane mixtures: DOPC–DOPS, DOPC–DOPA, and DOPC–DOPE were prepared with the CHARMM-GUI Membrane Builder online tool [32,33] with a molar composition of 4:1. The membrane models contain a total of 160 phospholipids and both the upper layer and lower layer have the same lipid molar composition.

A model structure for the NAF-1^44–67^, which we built in previous work [23], was inserted in the water region above the upper layer of the membrane. The system was solvated with TIP3P water molecules, and potassium and chloride ions were added to the solution giving a concentration of 150 mM.

All molecular simulations were performed with Gromacs (v.2019.4) [34] using the CHARMM 36 all-atom force field [35] and the CHARMM TIP3P water model [36]. Periodic boundary conditions were applied in all directions. The electrostatic interactions were calculated using the particle mesh Ewald (PME) method [37] with a real space cut-off of 1.2 nm and a mesh size of 0.12 nm. For the van der Waals interaction, a cut-off distance of 1.2 nm was used, and a switching term was added so the force smoothly decayed to zero from 1.0 to 1.2 nm. The systems were first energy-minimized using a steepest descent algorithm. After minimization, the systems were equilibrated for 375 ps using the default CHARMM membrane builder protocol, in which restraints of different molecular parts of the membrane system are gradually relaxed. Production simulations were run at the NPT ensemble with a Nosé–Hoover thermostat [38,39] at 323 K and a semi-isotropic Parrinello–Rahman barostat [40] at 1 bar. A time step of 2 fs was used for production runs. The SETTLE [41] and LINCS [42] algorithms constrained the water molecules and the bonds involving hydrogen atoms, respectively. Production runs were conducted for 3 μs, and the final 2 μs of the trajectories were used for analysis. These time scales are sufficiently long such that stable differences between the membranes can be detected. The time scales are not long enough to probe translocation of the peptide across the membrane, which can exceed seconds [23], but initial insertion to the upper layer of the membrane can be observed for all systems. To perform the analysis, we used Gromacs analysis tools [43] and Plumed [44]. For molecular visualization, we used VMD [45] and Chimera [46].

## 3. Results and Discussion

### 3.1. The Phospholipids

**Figure 2:**
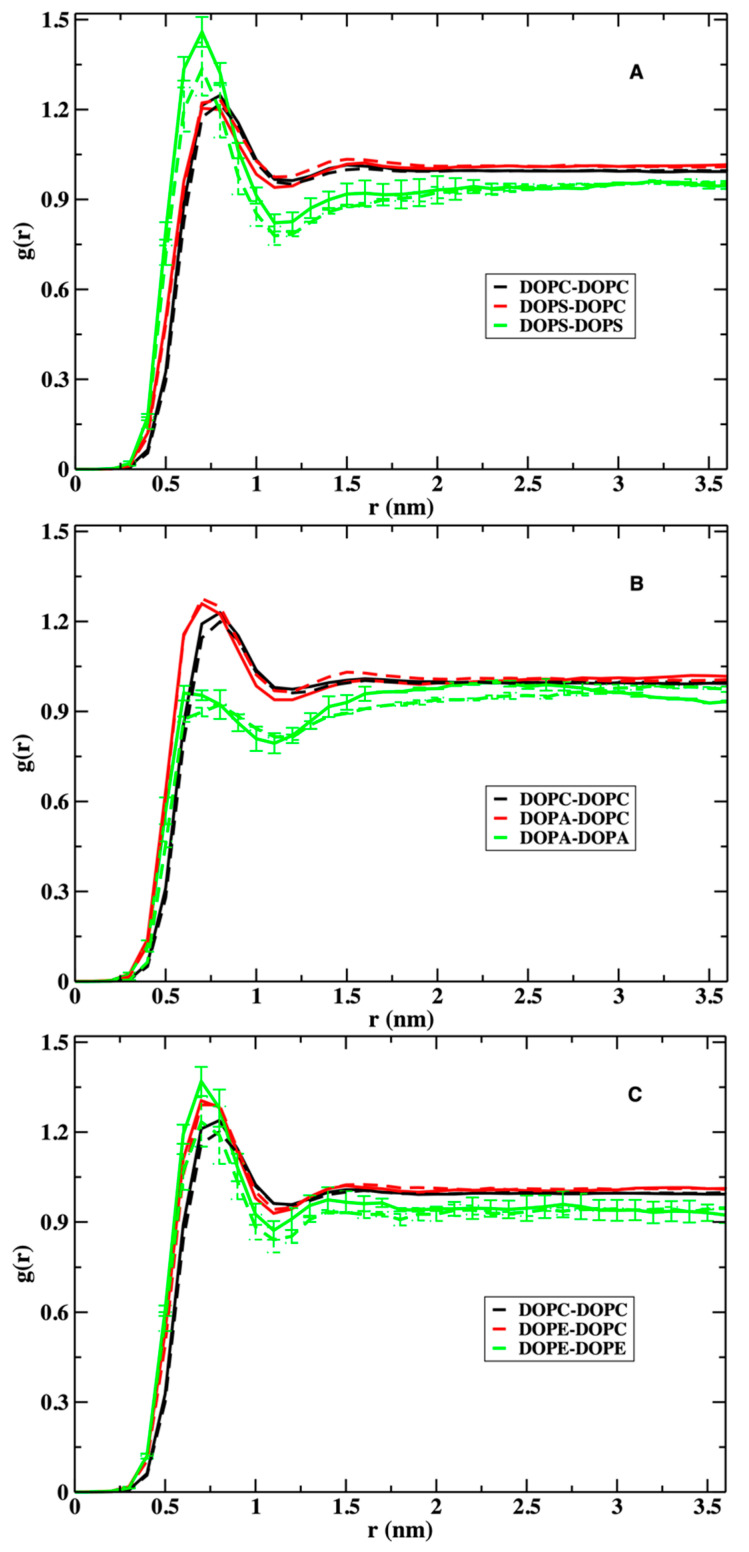
Radial distribution functions in the membrane plane of the phospholipid head groups in the three mixtures: panel (**A**) is for the membrane mixture of DOPC and DOPS, panel (**B**) is for DOPC–DOPA, and panel (**C**) is for DOPC–DOPE. The solid lines show the distributions at the upper layer of the membrane in which the peptide NAF-1^44–67^ is embedded and the dashed lines show the distributions at the lower layer. DOPS tends to attract other DOPS molecules. In contrast, DOPA molecules tend to repel each other. For clarity, the error bars (obtained by dividing the trajectory into two blocks) are only shown for the autocorrelations of DOPS, DOPA, and DOPE. The errors of the other correlations (not shown) are smaller.

In Figure 2, we show the distance correlation functions of the centers of mass of the head groups of the different phospholipids. We report the correlations separately for the upper and lower layers. The lower layer correlations are shown in dashed lines, while the upper layers are in solid lines. We show them both since the peptide is inserted to the upper layer, which makes the layers’ compositions asymmetric. Comparing the correlations in both layers provides information on the impact of NAF-1^44–67^ on the structure of the phospholipids. All correlations flattened out near 1.5 nm, illustrating their limited reach and the insensitivity of the distance range to the lipid composition or to the presence of the peptide.

The upper and lower layers have similar pair correlation functions at short distances, suggesting that the peptide perturbation to the overall interactions between the phospholipids is small. The only exception to this observation is the stronger correlation of DOPS–DOPS at the upper layer of the membrane. The largest errors in the pair correlation functions are ~0.1, suggesting that the differences between the upper and lower layers of DOPS–DOPS correlations are significant. The error bars were computed by dividing each of the 2 µs trajectories into two 1 µs trajectories and estimating the average and the errors from halves.

The phospholipids we examine differ in their overall charge and size of the head groups. Consider the uncharged phospholipids, DOPC and DOPE. The phospholipid DOPC is the major component in all membrane mixtures. Its distance distributions are roughly the same, regardless of the type of the other lipid or the presence of the peptide (Figure 2). The heights of DOPC first peaks are ~1.2 in the three panels. DOPE, the other neutral phospholipid, shows a more variable structure and has a higher first peak than that of DOPC in the presence of the peptide (~1.4, solid green line, Figure 2C). In the absence of the peptide, the first peak of DOPE is slightly reduced to ~1.2, the same height as that of DOPC. The smaller polar head of DOPE (NH_3_^+^) carries a higher charge density and is influenced by the charged peptide more than DOPC (N(CH_3_)_3_^+^) with a lower charge density (Figure 1). As a rule of thumb, a smaller head group implies stronger short-range interactions between the polar entities, which can be either attractive or repulsive. In the case of DOPE, it is attractive and leads to a higher first peak.

The correlation functions of the charged phospholipids are more complex. The self-correlations of DOPS and DOPA display opposite behaviors. The height of the first peak of DOPS–DOPS is close to 1.5 in the upper layer, indicating enrichment of DOPS in the neighborhood of the peptide. In contrast, DOPA distance correlations shows significant depletion at shorter distances with a peak height of about 0.9–1.0. This is perhaps not surprising given that the head groups that carry the same electric charges repel each other. However, it is less intuitive considering that DOPS–DOPS attract each other. The smaller head group of DOPA emphasizes direct interactions (in this case electrostatic repulsion), which lead to a reduction in short-range pairs compared to bulk. DOPS–DOPS charge repulsion is reduced by the bulkier head group and the pair is more comfortable in the presence of the positively charged peptide (Figure 2, panel A).

**Figure 3:**
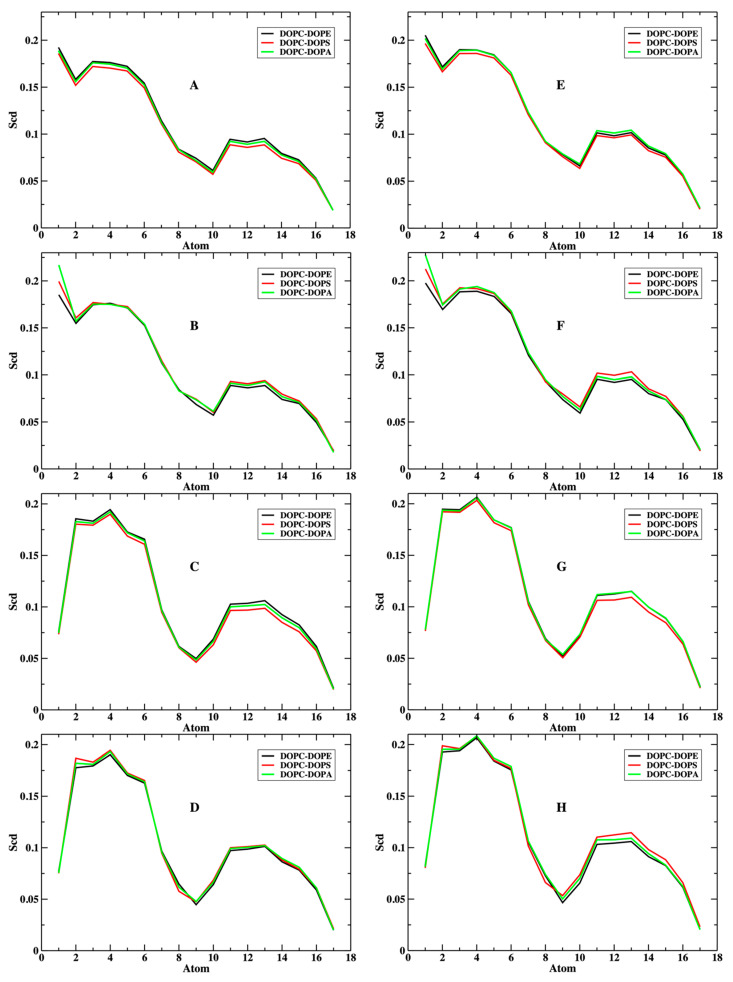
Deuterium order parameters for the lipid tails for the lower layers (**A**–**D**) and the upper layers of the membranes (**E**–**H**): (**A**,**E**) for the sn1 acyl chain of DOPC; (**B**,**F**) for the sn1 of the smaller component in the mixture; (**C**,**G**) for the sn2 acyl chain of DOPC; (**D**,**H**) for the sn2 chain of the lower component. Note the small differences between membranes of alternate mixtures. Note also that the upper layer (with the peptide) is slightly more ordered. The error bars are about the width of the lines in the plots and are not shown explicitly.

Another measure of the phospholipid structures is provided by the order parameter *S_CD_*; $S_{CD}=\frac{1}{2}\langle 3\cos^{2}\left(\theta \right)-1\rangle$ where the bracket 〈⋯〉 denotes ensemble averaging and the angle θ is between the C-D (in our case, C-H) bonds and the normal to the membrane. Perhaps the most remarkable observation from Figure 3 is the insensitivity of the order parameter to the phospholipid identity, at least on average. The phospholipids differ in their head groups but not in their tails, which partially explains these observations. The last differences do not seem to impact the orientational ordering of the hydrocarbon chains. The similarity of the plots between the upper and lower layers also demonstrates that the presence of the peptide NAF-1^44–67^ in the upper layer does not significantly impact the order parameters of the acyl chains.

### 3.2. The Peptide and the Phospholipids

**Figure 4:**
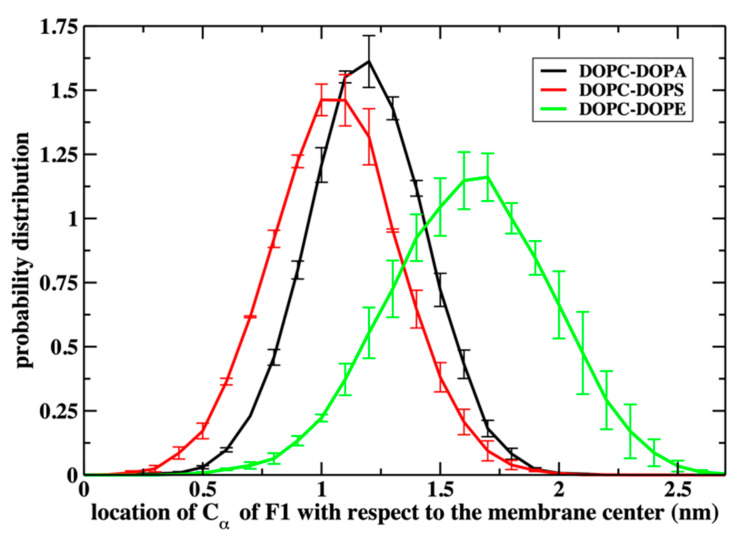
The distribution of the distances of the Cα of F1 from the membrane center. The N terminus segment of the peptide is in the hydrophobic region of the membrane for the two mixtures containing the negatively charged lipids (DOPS and DOPA). In contrast, the peak of the Cα distribution for DOPE–DOPC is just below the average phosphate location in the membrane (~1.8 nm). Error bars are obtained by dividing the trajectory data into two blocks.

In Figure 4, we show the distance of the alpha carbon of the first residue of the peptide (F1) from the center of the membrane. The first half of the peptide is primarily hydrophobic (Figure 1), permeating early into the membrane. The least significant permeation is to the uncharged membrane (DOPC–DOPE), presumably due to the resistance of the positively charged residues of the C terminus of the peptide to approach the hydrophobic core of the membrane. The zwitterionic phospholipids DOPC and DOPE are neutral, while DOPS and DOPA are negatively charged, reducing the impact of the peptide charges.

The difference between the permeation to the mixed membranes of DOPC–DOPA and DOPC-DOPS is relatively small. Hence, the headgroup charges significantly impact permeation even in the relatively short time scale of the simulations (μs). This observation about the impact of charges is similar to the assumed difference in NAF-1^44–67^ permeation to normal and malignant cells [22].

**Figure 5:**
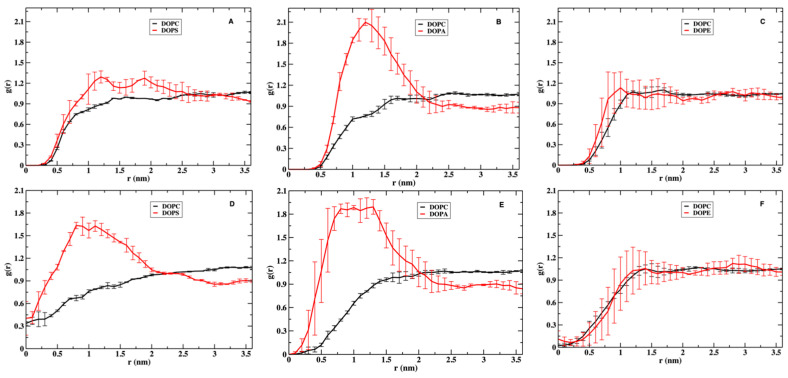
Radial distribution functions in the x–y plane of the membrane for the distances between NAF-1^44–67^ and the headgroups of the lipids in the three membrane mixtures. Panels (**A**–**C**) show the pair correlation functions of the phospholipid heads with the N terminus of the NAF-1^44–67^ peptide (residues 1 to 11) and panels (**D**–**F**) with the C terminus of the peptide (residues 12 to 24). Note the significant first peak in the distributions of the proximate DOPA, both to the C and N terminus. In contrast, DOPS’s proximate presence is large only near the C terminus of NAF-1^44–67^. Note also that the C terminal can be off the membrane plane; therefore, the distances may not reach zero (e.g., panel **D**). Error bars are obtained by dividing the trajectory data into two blocks.

Further analysis of the interactions of the peptide with charged and uncharged phospholipids is provided in Figure 5. We show the distributions of the lateral distances between the head groups of the phospholipids and the center of masses of the N and C terminal segments of the NAF-1^44–67^ (residues 1 to 11 for the N terminus and residues 12 to 24 for the C terminus). In panels C and F, we illustrate that the neutral phospholipids do not form significant structure near the peptide. Excluded volume prevents the peptide and the DOPC or DOPE molecules from approaching closer than ~0.5 nm. However, beyond the excluded volume, the distributions are flat, indicating a lack of long-range correlation.

More structure is observed in panel A, in which the charged phospholipid DOPS is closer to the peptide than the neutral DOPC. Significant first-neighbor peaks are shown in panels B, D, and E. The presence of charged phospholipids, DOPA, and DOPS is significantly enriched near the peptide compared to the bulk distribution. The phospholipid with the smaller head, DOPA, is found more frequently close to the N and the C termini. DOPS has a larger head, and its presence is significantly enriched compared to the bulk distribution near the C terminus. The C terminal segment carries the peptide charges. Hence, the specific rearrangements of the membranes’ phospholipids near NAF-1^44–67^ are a significant deviation from a uniform flat (bulk) distribution. The organization near the peptide can be considered a microdomain enriched with charged phospholipids such as DOPA or DOPS. It can be classified, perhaps, as an example of a small raft [47].

**Figure 6:**
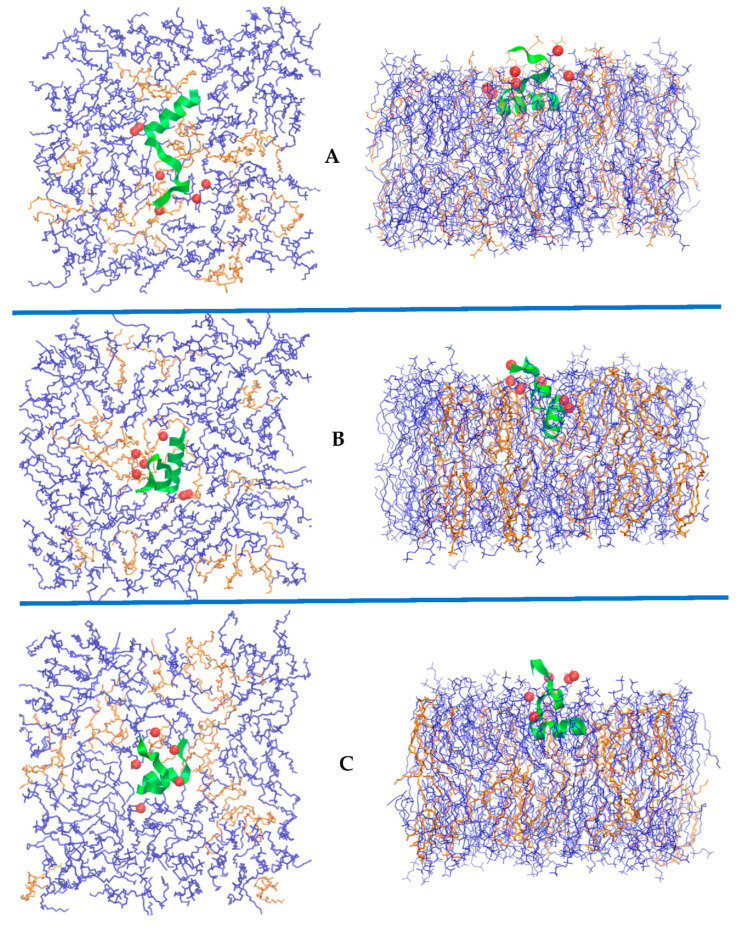
Top and side view configurations of NAF-1^44–67^ peptide inserted into the membrane systems: (**A**) DOPC–DOPS; (**B**) DOPC–DOPA; (**C**) DOPC–DOPE. In all the images, the DOPC molecules are shown in blue and the other lipid is in orange. Green ribbons are used to show NAF-1^44–67^ with the positive charges of the lysine and arginine residues shown with red van der Waals spheres. Water molecules are not shown but are included in the simulations. For clarity, the top views show the lipids only in the upper layer of the membranes.

**Figure 7:**
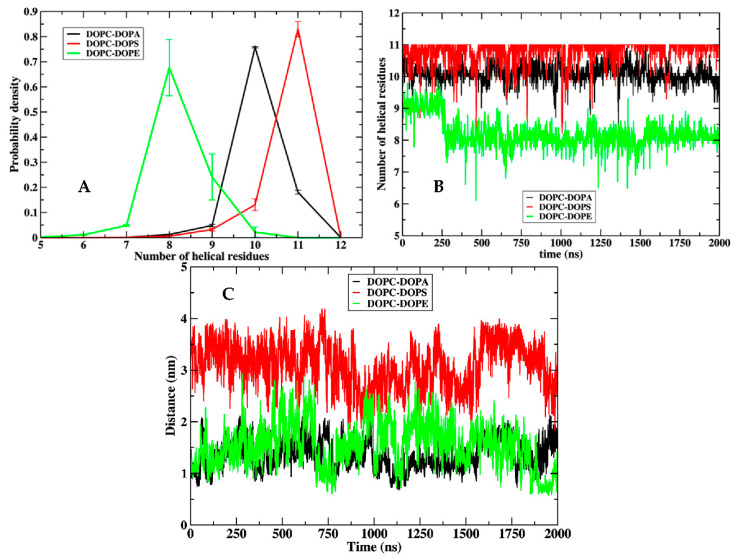
(**A**): The probability density for the number of residues in a helical state for NAF1^44–67^ for the three different membrane mixtures. The highest content of helical residues is for the DOPC–DOPS membrane. The smallest helical content is in the membrane mixture of DOPC–DOPE as is also observed in Figure 6. Error bars are obtained by dividing the trajectory data into two blocks. (**B**) Changes in the helical content of NAF1^44–67^ for the last 2 μs of the trajectories for the three membrane systems. The helical N terminus is inserted in the hydrophobic region of the membrane for the DOPC–DOPA and DOPC–DOPS systems, but not for the DOPC-DOPE membrane (see Figure 4). (**C**) The distance between the center of mass of the C terminal and the N terminal segments of the peptide as a function of time in the three membranes. This distance provides a measure of the peptide compactness.

In Figure 6, we display molecular dynamics snapshots of the peptide embedded in the membranes. For clarity, the water molecules and solvating ions are not shown. The charges of the peptide (red spheres) remain close to the membrane surface. As we indicated in our previous studies [23,48,49,50], significant membrane distortions are required to allow the charges to pass the hydrophobic core and to switch to the other side of the membrane. These events did not occur in the relatively short simulations we conducted here. Visual inspection (Figure 6, panel B) supports the increase in the number of DOPA molecules near the peptide, as suggested in Figure 5, panels B and E. Overall, the peptide retains the helical structure of the N terminus throughout the simulations (Figure 7, panels A and B) and the helix axis is parallel to the membrane surface. The hydrophobic helix is less stable in an aqueous solution, and its retention depends on permeation to the membrane. In the DOPC–DOPE membrane, the peptide permeation is less deep, and the helical content is reduced (Figure 7, panels A and B). In contrast, DOPC–DOPS and DOPC–DOPA, both charged membranes, are more successful in preserving the helical segment. Figure 7A,B shows that the helix is best retained in the DOPC–DOPS mixture. This is perhaps due to the amphiphilic peptide. The hydrophobic part is well solvated in the lipid and the hydrophilic part in an aqueous solution. The binary mixture of DOPC–DOPS successfully separates the two segments (Figure 7, panel C), leading to efficient initial permeation.

**Figure 8:**
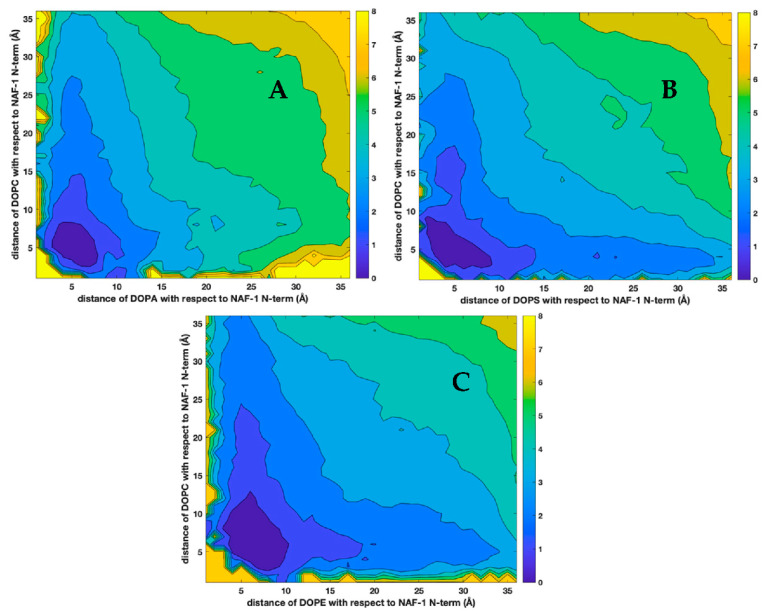
Distributions of pairs of distances between different phospholipids and the NAF-1^44–67^ peptide. (**A**) DOPA–DOPC, (**B**) DOPS–DOPC, (**C**) DOPE–DOPC. See text for more details.

**Figure 9:**
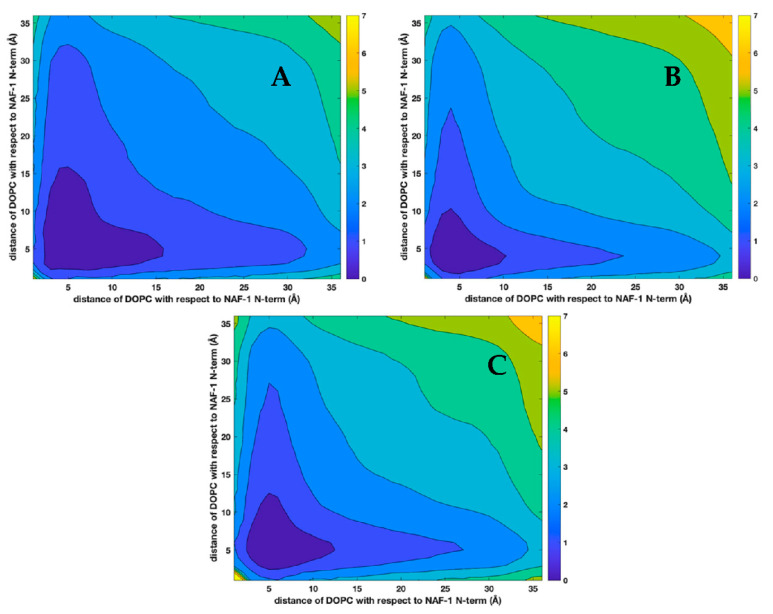
The self-distance correlations between the center of mass of the N-terminus of NAF-1^44–67^ and the center of mass of DOPC computed in three dimensions for the different lipid mixtures. The panels are for the three membranes: (**A**) DOPC–DOPA, (**B**) DOPC–DOPS, and (**C**) DOPC–DOPE.

Membranes are highly viscous fluids with strong interactions between their components and significant dynamic and static correlations. The findings of the formation of spatial and temporal microdomains in membranes demonstrate those correlations [10,11,49,51,52]. To examine correlations beyond pairs (Figure 5) we plotted distributions of three body distances from the phospholipids to the center of mass of the peptide (Figure 8 and Figure 9).

There are four times more DOPC molecules than other lipids in the mixtures. The distributions, even with a lack of interactions, reflect the larger number of DOPC molecules in the systems. Indeed, all the panels in Figure 8 suggest that far away from the peptide (e.g., distances of 20 Å), there is a higher probability of observing a DOPC molecule than the other lipid. Moreover, the overall shape of the contour lines tends to follow the axes and suggests a lack of correlation between the two distances from the phospholipids to the peptides. Since the fraction of all other lipids is the same (20 percent), comparing the panels can further assess the attraction between the phospholipid and the peptide and supplants Figure 5. DOPA is found mostly near the peptide, while DOPS and DOPE are more likely to be found at larger distances.

In Figure 9, we show the self-correlation of the distances of DOPC from the peptide in the three membranes. Panel (A) is for the mixture of DOPC and DOPA, panel (B) for DOPC–DOPS, and panel (C) for DOPC–DOPE. Panels (B) and (C) are quite similar, suggesting similar membrane–peptide interactions for DOPC–DOPS and DOPC–DOPE, regardless of the molecular differences (i.e., DOPS is charged and DOPE is not). In contrast, DOPA, which is charged, is enriched with DOPC in a larger area than the two other mixtures. This enrichment happens because DOPA is closer to the peptide (Figure 8, panel A) Note that the correlation plots are computed in three dimensions, and binning is over two different spherical shells, so it supplants the two-dimensional analysis of Figure 5.

## 4. Conclusions

We have illustrated that alternate heterogeneous membranes interact differently with a novel anticancer peptide. The peptide is derived from a transmembrane protein and includes a purely hydrophobic N terminal segment and a significantly charged C terminus (+5). The hydrophobic part permeates first, overcoming a head-group barrier, as we illustrated in a recent paper [23]. Therefore, variations in the composition of the head groups are of significant interest. We identified the strong reorganization of the phospholipid distribution near the permeating peptide. The preferences can be interpreted as microdomains (or rafts) [10]. Overall, the peptide’s perturbation on the phospholipid characteristic is minimal. No significant reordering of the hydrocarbon chains was observed as probed by the *S_CD_* order parameters. Furthermore, we did not detect large-scale correlations between the phospholipid molecules with or without the peptide. Hence, a reasonable picture of the peptide–membrane interaction is of short-range adjustments of phospholipid compositions near the peptide and the loss of significant correlations beyond the first interaction shell. In the future, it will be interesting to consider more complex mixtures and other inserts to the membrane, such as cholesterol.

Based on the permeation selectivity of the different membranes, the membrane that most resembles the plasma membranes of normal cells is DOPC–DOPE. This neutral binary membrane is the least permeable. DOPC–DOPS, which is charged, better separates the two segments of the amphiphilic peptide and supports initial permeation; this resembles the plasma membrane of cancer cells.

## Data Availability

Not applicable.
